# The Role of Screening and Treatment in National Progress Toward Hepatitis C Elimination — Georgia, 2015–2016

**DOI:** 10.15585/mmwr.mm6629a2

**Published:** 2017-07-28

**Authors:** Muazzam Nasrullah, David Sergeenko, Lia Gvinjilia, Amiran Gamkrelidze, Tengiz Tsertsvadze, Maia Butsashvili, David Metreveli, Lali Sharvadze, Maia Alkhazashvili, Shaun Shadaker, John W. Ward, Juliette Morgan, Francisco Averhoff

**Affiliations:** ^1^Division of Viral Hepatitis, National Center for HIV/AIDS, Viral Hepatitis, STD and TB Prevention, CDC; ^2^Ministry of Labor Health and Social Affairs of Georgia; ^3^CDC Foundation; ^4^National Center for Disease Control and Public Health of Georgia; ^5^Infection Diseases, AIDS, and Clinical Immunology Research Center, Tbilisi, Georgia; ^6^Neolab, Tbilisi, Georgia; ^7^Medical Center Mrcheveli, Tbilisi, Georgia; ^8^Joint Georgian-French Hepatology Clinic Hepa, Tbilisi, Georgia; ^9^Global Disease Detection, Division of Global Health Protection, South Caucasus CDC Office, Tbilisi, Georgia; ^10^Division of Global Health Protection, Center for Global Health, CDC.

Georgia, a country in the Caucasus region of Eurasia, has a high prevalence of hepatitis C virus (HCV) infection. In April 2015, with technical assistance from CDC, Georgia embarked on the world’s first program to eliminate hepatitis C, defined as a 90% reduction in HCV prevalence by 2020 (*1*,*2*). The country committed to identifying infected persons and linking them to care and curative antiviral therapy, which was provided free of charge through a partnership with Gilead Sciences (*1*,*2*). From April 2015 through December 2016, a total of 27,595 persons initiated treatment for HCV infection, among whom 19,778 (71.7%) completed treatment. Among 6,366 persons tested for HCV RNA ≥12 weeks after completing treatment, 5,356 (84.1%) had no detectable virus in their blood, indicative of a sustained virologic response (SVR) and cure of HCV infection. The number of persons initiating treatment peaked in September 2016 at 4,595 and declined during October–December. Broader implementation of interventions that increase access to HCV testing, care, and treatment for persons living with HCV are needed for Georgia to reach national targets for the elimination of HCV.

In 2015, an estimated 5.4% of the adult population of Georgia (approximately 150,000 persons) had chronic HCV infection, and of those, nearly two thirds were unaware of their infection (Georgia Ministry of Labour, Health, and Social Affairs [MoLHSA], unpublished data, 2016). Populations with the highest rates of HCV infection include men, persons aged 30–59 years, persons with a history of injection drug use, and persons with a history of receipt of blood products (MoLHSA, unpublished data, 2016). Initially, when the program was launched in April 2015, national guidelines limited treatment to HCV-infected persons with advanced liver disease, defined as one or both of the following: F3 or F4 by METAVIR fibrosis score (a system used to assess the histological extent of hepatic inflammation and fibrosis in patients with hepatitis C infection) on transient elastography or FIB-4 score (a noninvasive test based on a combination of biochemical values and patient age) >3.25 (*3*,*4*). In June 2016, treatment eligibility criteria were expanded to include all HCV-infected persons, regardless of disease severity.

HCV screening programs began in January 2015, before the launch of the program, and screening services continue to be provided at various settings at no cost (Table). During January 2015–December 2016, a total of 472,890 HCV screening tests* were conducted, 50,962 (10.8%) of which were positive for HCV antibody. The highest rate of HCV antibody–positive screening tests (45.0%) was among persons who attended programs providing services for persons who inject drugs; the lowest rate (0.4%) was among women attending antenatal clinics (Table). Persons who screen positive for HCV antibody are referred to the treatment program for confirmation of chronic HCV infection using polymerase chain reaction (PCR) testing for detection of HCV RNA. Once chronic HCV infection is confirmed, the person is invited to enroll in the treatment program.

**TABLE:** Number of screening tests* for hepatitis C virus (N = 472,890) and percentage testing positive, by group screened — Georgia, 2015–2016

Group screened/Location of screening	No. screening tests	% HCV positive
Blood donors	168,121	1.3
NCDC	83,910	17.5
Pregnant women/ANCs	53,852	0.4
Hospitalized patients^†^	48,025	4.9
Persons who inject drugs	44,410	45.0
Tblisi citizens^§^	26,159	13.8
Outpatients^†^	18,900	7.4
Prisoners	14,053	37.4
Military recruits	11,217	1.5
HCV screening or treatment center	2,453	31.4
Persons living with HIV	1,790	24.9
**Total**	**472,890**	**10.8**

**Abbreviations**: ANC = antenatal clinic; HCV = hepatitis C virus; HIV = human immunodeficiency virus; NCDC = National Centers for Disease Control and Public Health headquarters and regional centers.

* Number of HCV screening tests (not individual persons) reported to NCDC.

^†^ Data are from November 1–December 30, 2016.

^§^ Screening centers operated by the city of Tbilisi.

When the treatment program began on April 28, 2015, four treatment centers operated in Georgia, all located in Tbilisi, the capital and largest city. By December 2016, the number of treatment centers had increased to 27 nationwide. From the start to December 31, 2016, a total of 58,223 persons with positive HCV antibody test results sought confirmation of chronic HCV infection through the treatment program, among whom 38,113 (65.5%) initiated a diagnostic evaluation, including confirmation of HCV infection by PCR testing; of those who initiated a diagnostic evaluation, 30,046 (78.8%) were confirmed as having chronic HCV infection and completed the diagnostic workup, and 27,595 (91.8%) of whom began treatment. Men accounted for 23,062 (83.6%) of all persons starting treatment, including 9,180 men aged 40–49 years, representing one third of all persons who initiated treatment (Figure 1). The average number of persons starting treatment each month increased nearly 300% from April 2015–May 2016 (661 per month) to June–December, 2016 (2,619 per month), peaking in September 2016 at 4,595. A decline occurred from October through December 2016 (Figure 2). During the initial phase of the program (April, 2015–May, 2016), when treatment was prioritized for persons with more severe liver disease, most patients initiating treatment (9,088 of 9,259; 98.2%) had advanced liver disease (≥F3 METAVIR fibrosis score or FIB-4 score >3.25). After the expansion of treatment criteria to allow treatment for all persons with HCV infection (beginning June 1 through December 31, 2016), most persons initiating treatment (14,368 of 18,336; 78.4%) had less severe liver disease (<F3 METAVIR fibrosis score or FIB-4 score <1.45) (Figure 2).

**FIGURE 1:**
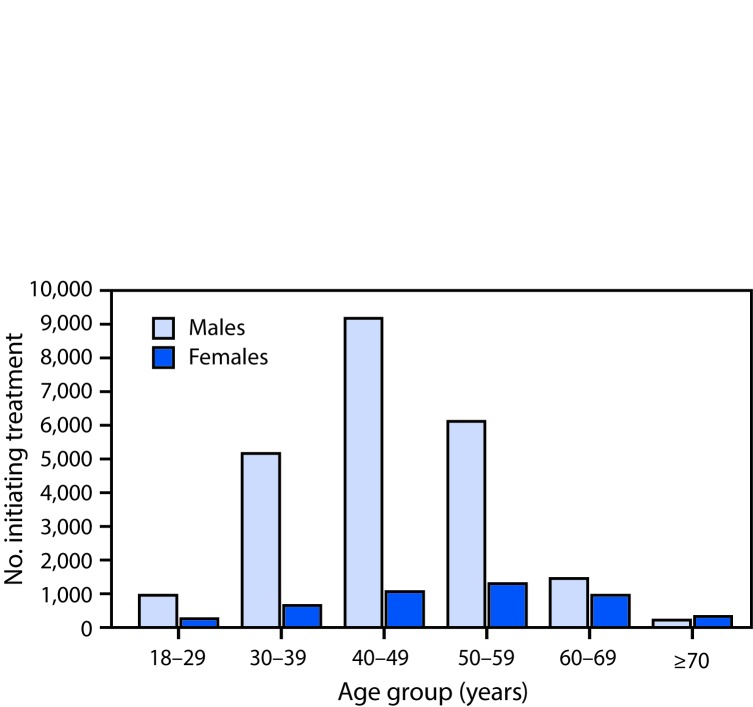
Number of persons initiating treatment for hepatitis C virus infection, by sex and age group — Georgia, April 2015–December 2016* * The age group “18–29 years” includes five female patients aged 13–17 years.

**FIGURE 2:**
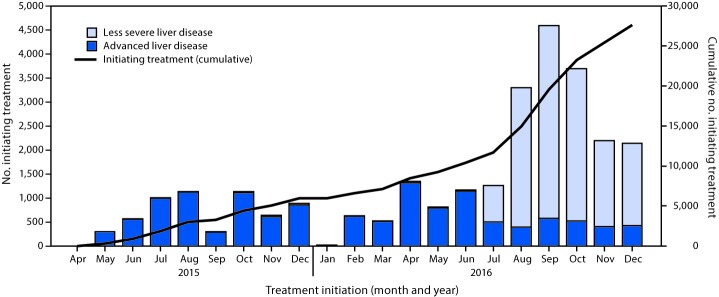
Number of persons initiating treatment for hepatitis C virus infection and cumulative number initiating treatment, by severity of liver disease* and month — Georgia, April 2015–December 2016 * Less severe liver disease defined as <F3 METAVIR fibrosis score and/or FIB-4 score <1.45; advanced liver disease defined as ≥F3 METAVIR fibrosis score and/or FIB-4 score >3.25.

As of December 31, 2016, a total of 19,778 persons completed treatment, and 6,366 (32.2%) eligible patients received testing for SVR (undetectable HCV RNA ≥12 weeks after treatment completion) (*5*). SVR was observed for 5,356 (84.1%) persons tested, indicating that they were cured of their infection. Among the 75.0% (4,774/6,366) who received sofosbuvir (without ledipasvir) treatment regimens, 3,793 (79.5%) achieved SVR, and among the 25.0% (1,592 of 6,366) who received ledipasvir/sofosbuvir-based treatment regimens, 1,563 (98.2%) achieved SVR. Among 537 (1.9%) persons who did not complete treatment, 371 (69.1%) died from their liver disease or another cause during the course of treatment, and the other 166 (30.1%) discontinued treatment for other reasons. 

## Discussion

Since the launch of the Georgia HCV Elimination Program in April 2015, progress has been made in providing treatment to and curing persons infected with HCV, including a 300% increase in the average monthly number of patients initiating treatment during the second half of 2016. These gains are attributed to an increase in the number of treatment sites, expansion of treatment eligibility criteria, and introduction of a newer, highly effective all-oral combination antiviral drug (ledipasvir/sofosbuvir) (*6*). However, enrollment in the treatment program declined considerably during the last 3 months of 2016. This decline is likely because of patients’ lack of awareness of their infection status or lack of access to the treatment program for HCV-infected persons who were aware of their infection. The data in this report suggest that a substantial proportion of persons tested and found positive for HCV antibodies are not successfully referred for evaluation of HCV infection. Through December 2016, approximately 20% of the estimated 150,000 Georgians living with HCV infection entered the treatment program. Increased measures to identify infected persons and link them to care and treatment are needed to reach the 2020 elimination goal of 90% reduction in HCV prevalence.

At the launch of the program in 2015, national serologic survey data revealed about one third of HCV-infected Georgians were aware of their infection (MoLHSA, unpublished data, 2016). Data are lacking on how many of the approximately 51,000 persons who screened positive for HCV during 2015 and 2016 accessed the program to receive confirmatory testing (which unlike initial screening, is not free of charge) and entered the treatment program if chronic HCV infection was confirmed. Changes in government policies that target large at-risk populations, offer free HCV confirmatory testing and additional diagnostic evaluation for patients with confirmed HCV infection, increase the number of providers that can provide testing and treatment services, and support campaigns to expand public awareness and demand for HCV services can increase HCV screening and treatment rates.

Although approximately 470,000 HCV screening tests were reported during 2015–2016, many at-risk Georgians remain unscreened. HCV prevalence varied markedly across different screening settings and programs: screening conducted at antenatal clinics yielded a low proportion of persons screening positive, and screening at corrections and harm-reduction facilities yielded high HCV prevalence rates. Targeted provision of testing and linkage to care services might increase the detection of persons with HCV infection, and thereby, the number entering the treatment program.

Reaching the 2020 HCV elimination goals will require innovative strategies to increase awareness, expand access to high-quality screening, and remove diagnostic and treatment barriers which may include costs associated with confirmatory testing and diagnostic workup, stigma, and distance to treatment centers. Increased impact can be achieved by providing services at primary care settings and settings serving populations at high risk (e.g., syringe service programs for injection drug users).

Elimination of HCV infection in Georgia hinges not only on strategies that identify, treat, and cure persons of their infection, but also on those that prevent new infections. To ensure a comprehensive approach to HCV elimination, MoLHSA developed a *Strategic Plan for Elimination of Hepatitis C in Georgia* (*7*). In addition to proposing actions to improve HCV screening and linkage to care, the plan identifies strategies for preventing new infections, including improving safety of the blood supply, ensuring infection control in health care settings, and providing persons who inject drugs with harm-reduction services.

The findings in this report are subject to at least three limitations. First, data from the screening and treatment programs could not be independently verified and might be subject to data entry errors. Second, the screening data reported might include persons who received repeat testing; thus it is not known whether each HCV antibody test represents a single person screened. Finally, HCV screening data are not linked to treatment data, and as a result, this analysis could not assess the effectiveness of linkage of screening to the care and treatment program.

Despite notable progress during the first 20 months of the Georgia HCV elimination program, challenges to Georgia achieving the national targets for HCV elimination by 2020 remain. High-quality screening, innovative linkage-to-care strategies, and cost-effective and simplified diagnostic and treatment regimens are needed. Provision of free-of-charge services for HCV screening, diagnosis, care, and treatment in settings serving populations at high risk for HCV infection and in primary care settings can decrease barriers to access of treatment services. MoLHSA is working with CDC and other international partners to address challenges and introduce innovative strategies. Pangenotypic direct-acting antiviral drugs that are effective across the different genotypes of HCV, point-of-care HCV RNA testing, and HCV core antigen testing are likely to be introduced in late 2017 or 2018 and could have a substantial impact on improving access and simplifying diagnosis and treatment. Information systems capable of linking screening and treatment data are being developed to improve efficiencies. With increased access to HCV treatment services and full implementation of the country’s strategic plan, Georgia can achieve the goal for HCV elimination in 2020. Lessons learned from this program can inform similar initiatives in other countries and help curb the global epidemic of viral hepatitis (*8*).

**Summary**

**What is already known about this topic?**

An estimated 150,000 persons in the country of Georgia (5.4% of the adult population) are infected with hepatitis C virus (HCV). In April 2015, in collaboration with CDC and other partners, Georgia launched a program to eliminate HCV by 2020. An important strategy is the identification of HCV-infected persons and provision of curative antiviral therapy.

**What is added by this report?**

During April 28, 2015–December 31, 2016, a total of 27,595 HCV-infected persons started therapy, 19,778 (71.7%) of whom completed treatment. Among 6,366 (32.2%) who completed treatment and were tested for treatment response, 5,356 (84.1%) were cured of their HCV infection. The average number of persons who initiated treatment each month increased threefold from April 2015–May 2016, when treatment was limited to persons with severe liver disease, to June–December 2016, after expansion of the eligibility criteria to allow treatment of all HCV-infected persons. During the last 3 months of 2016, the number of persons entering the treatment program declined steadily, suggesting that identification and linkage to care of HCV infected persons in the country might be slowing.

**What are the implications for public health practice?**

The Georgia HCV Elimination Program has made substantial progress since its launch in April 2015; the country has demonstrated the ability to scale up HCV care and treatment services rapidly. Enhancing HCV testing and linkage to care and treatment services are critical to reaching the 2020 HCV elimination goal. Lessons learned from the Georgia elimination program can inform programs in other countries striving to eliminate HCV as a public health threat.

## Footnotes

* Hepatitis C virus rapid tests by all screening programs except blood banks that mostly used enzyme immunoassay.

## References

[1] Mitruka K, Tsertsvadze T, Butsashvili M, Launch of a nationwide hepatitis C elimination program—Georgia, April 2015. MMWR Morb Mortal Wkly Rep 2015;64:753–7. 10.15585/mmwr.mm6428a2 26203628 PMC4584859

[2] Gvinjilia L, Nasrullah M, Sergeenko D, National progress toward hepatitis C elimination—Georgia, 2015–2016. MMWR Morb Mortal Wkly Rep 2016;65:1132–5. 10.15585/mmwr.mm6541a2 27764081

[3] Bedossa P, Poynard T; The METAVIR Cooperative Study Group. An algorithm for the grading of activity in chronic hepatitis C. Hepatology 1996;24:289–93. 10.1002/hep.510240201 8690394

[4] Vallet-Pichard A, Mallet V, Nalpas B, FIB-4: an inexpensive and accurate marker of fibrosis in HCV infection. comparison with liver biopsy and fibrotest. Hepatology 2007;46:32–6. 10.1002/hep.21669 17567829

[5] Wedemeyer H, Jensen DM, Godofsky E, Mani N, Pawlotsky VM, Miller V; Definitions/Nomenclature Working Group of the HCV DrAG (HCV Drug Development Advisory Group), under the auspices of the Forum for Collaborative HIV Research. Recommendations for standardized nomenclature and definitions of viral response in trial of hepatitis C virus investigational agents. Hepatology 2012;56:2398–403. 10.1002/hep.25888 22707382

[6] Pawlotsky JM, Feld JJ, Zeuzem S, Hoofnagle JH. From non-A, non-B hepatitis to hepatitis C virus cure. J Hepatol 2015;62(Suppl):S87–99. 10.1016/j.jhep.2015.02.006 25920094

[7] Ministry of Labour Health and Social Affairs of Georgia. Strategic plan for the elimination of hepatitis C virus in Georgia, 2016–2020. Tbilisi, Georgia: Ministry of Labour Health and Social Affairs; 2017. http://moh.gov.ge/ka/528/

[8] World Health Organization. Global hepatitis report, 2017. Geneva, Switzerland: World Health Organization; 2017. http://apps.who.int/iris/bitstream/10665/255016/1/9789241565455-eng.pdf?ua=1

