# *Erratum*: Vol. 66, No. 29

**DOI:** 10.15585/mmwr.mm6631a8

**Published:** 2017-08-11

In the announcement on World Hepatitis Day on page 794, the title should have read “World Hepatitis Day — **July 28**, 2017.”

